# Evolutionary history and functional divergence of the cytochrome P450 gene superfamily between *Arabidopsis thaliana* and *Brassica* species uncover effects of whole genome and tandem duplications

**DOI:** 10.1186/s12864-017-4094-7

**Published:** 2017-09-18

**Authors:** Jingyin Yu, Sadia Tehrim, Linhai Wang, Komivi Dossa, Xiurong Zhang, Tao Ke, Boshou Liao

**Affiliations:** 1 Key Laboratory of Biology and Genetic Improvement of Oil Crops, Ministry of Agriculture, Oil Crops Research Institute, the Chinese Academy of Agricultural Sciences, Wuhan, 430062 China; 2 Centre d’Etudes Régional pour l’Amélioration de l’Adaptation à la Sécheresse (CERAAS), BP 3320 Route de Khombole, Thiès, Sénégal; 3 Department of Life Science and Technology, Nanyang Normal University, Wolong Road, Nanyang, 473061 China

**Keywords:** Cytochrome P450s, *Brassica*, Tandem duplication, Whole genome duplication, Evolution mechanism, Expression profiling

## Abstract

**Background:**

The cytochrome P450 monooxygenase (P450) superfamily is involved in the biosynthesis of various primary and secondary metabolites. However, little is known about the effects of whole genome duplication (WGD) and tandem duplication (TD) events on the evolutionary history and functional divergence of P450s in *Brassica* after splitting from a common ancestor with *Arabidopsis thaliana*.

**Results:**

Using Hidden Markov Model search and manual curation, we detected that *Brassica* species have nearly 1.4-fold as many P450 members as *A. thaliana*. Most P450s in *A. thaliana* and *Brassica* species were located on pseudo-chromosomes. The inferred phylogeny indicated that all P450s were clustered into two different subgroups. Analysis of WGD event revealed that different P450 gene families had appeared after evolutionary events of species. For the TD event analyses, the P450s from TD events in *Brassica* species can be divided into ancient and recent parts. Our comparison of influence of WGD and TD events on the P450 gene superfamily between *A. thaliana* and *Brassica* species indicated that the family-specific evolution in the *Brassica* lineage can be attributed to both WGD and TD, whereas WGD was recognized as the major mechanism for the recent evolution of the P450 super gene family. Expression analysis of P450s from *A. thaliana* and *Brassica* species indicated that WGD-type P450s showed the same expression pattern but completely different expression with TD-type P450s across different tissues in *Brassica* species. Selection force analysis suggested that P450 orthologous gene pairs between *A. thaliana* and *Brassica* species underwent negative selection, but no significant differences were found between P450 orthologous gene pairs in *A. thaliana*–*B. rapa* and *A. thaliana*–*B. oleracea* lineages, as well as in different subgenomes in *B. rapa* or *B. oleracea* compared with *A. thaliana*.

**Conclusions:**

This study is the first to investigate the effects of WGD and TD on the evolutionary history and functional divergence of P450 gene families in *A. thaliana* and *Brassica* species. This study provides a biology model to study the mechanism of gene family formation, particularly in the context of the evolutionary history of angiosperms, and offers novel insights for the study of angiosperm genomes**.**

**Electronic supplementary material:**

The online version of this article (10.1186/s12864-017-4094-7) contains supplementary material, which is available to authorized users.

## Background

The cytochrome P450 monooxygenase (P450) superfamily comprises heme-thiolate proteins that are mostly attached to the cytoplasmic surface of the endoplasmic reticulum and are involved in different NADPH- and O_2_-dependent hydroxylation reactions [1]. P450 proteins serve diverse biological functions in plants. Many P450s are involved in the syntheses of pigments, antioxidants, structural polymers, and defense-related compounds, such as flavonoids, phenylpropanoids, phenolic esters, coumarins, alkaloids, terpenoids, lipids, cyanogenic glycosides, glucosinolates, benzoxazinones, and isoprenoids, whereas others are crucial for the synthesis and catabolism of plant growth regulators and signaling molecules. Similar to their animal counterparts, some P450s are also involved in the detoxification of pollutants, herbicides, and insecticides [2].

The first cytochrome P450 gene was found in the rat liver 56 years ago [3]. Subsequently, it was identified in all domains of life, such as plants, mammals, fish, insects, fungi, bacteria, and viruses. To date, more than 18,500 sequences of P450s have been reported from hundreds of species [4]. The universal guidelines for the nomenclature of P450 genes suggest that a newly discovered P450 protein sharing a sequence similarity greater than 40% to a previously identified P450 family should be grouped into the same family but should be classified under a new family when the sequence similarity is 40% or less. Similarly, same-family P450s with a sharing identity greater than 55% are grouped in the same subfamily but are separated when the sharing identity is less than 55%. In addition, P450s with a sharing identity of 97% or more are considered allelic variants of the same gene, and genes encoding the same P450 protein from different species are assigned to the same subfamily [5, 6]. According to the evolutionary relationship of P450s in different organisms, plant P450s are generally categorized into two groups: A type and non-A type. The first category includes P450s that are specific to plants and have evolved since the divergence of plants from animals and fungi; the second category includes non-plant-specific P450s that share the sequence similarity to animals and fungal enzymes [7]. Nowadays, in the current plant P450 phylogeny, plant P450 families are grouped into nine different clans, including CYP51, CYP71, CYP710, CYP711, CYP72, CYP74, CYP85, CYP86, and CYP97 clans [8]. CYP 71 clan was classified into A type P450s, and the remaining eight clans were classified into non-A type. These analyses brought critical information for studying the phylogeny of cytochrome P450 gene family.

So far, the available genome sequences in plants have provided more than 3000 P450 genes, making them a vast gene superfamily in plant genomes. For instance, previous studies reported approximate arrays of 356 cytochrome P450 genes in *Oryza sativa*, 312 in *Populus trichocarpa*, 457 in *Vitis vinifera*, 39 in *Chlamydomonas reinhardtii*, 71 in *Physcomitrella patens*, 225 in *Selaginella moellendorffii*, and 313 in *Lotus japonicas* [6, 9, 10]. The number of cytochrome P450 gene datasets is expected to increase with the development of genome sequencing technologies, which will allow researchers to study these genes from the perspective of comparative genomics or evolutionary biology.

**Fig. 1:**
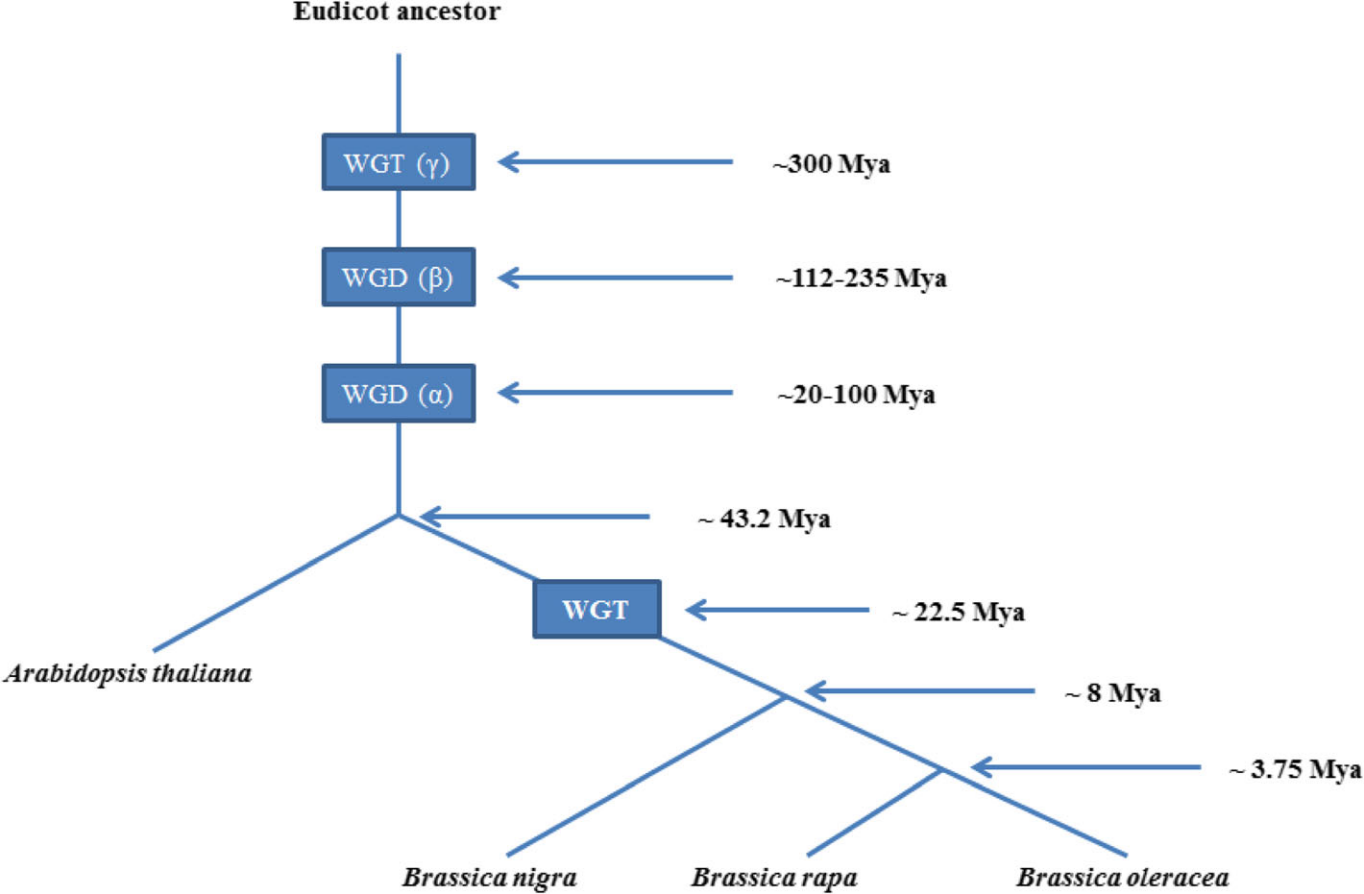
Ancestral polyploidy events and corresponding occurrence times in eudicots. Blue rectangles represent whole genome duplication or whole genome triplication events

During the past decade, the model plant *Arabidopsis* from the *Brassicaceae* family has broadened the understanding of plant biology, especially genome evolution in angiosperms. Evolutionary patterns suggest the occurrence of three rounds (i.e., γ, β, and α) of whole genome duplication or triplication (WGD or WGT) in *A. thaliana* and all *Brassica*ceae taxa [11]. The first WGT (γ) has been linked to the diversification of eudicots and angiosperms [12, 13].The second duplication (β), which was estimated to have occurred between 235 and 112 Mya [11, 14, 15]. The most recent α duplication occurred between 100 and 20 Mya [11, 16]. After the α duplication, Arabidopsis lineage separated from its *Brassica* ancestor approximately 43.2 MYA, and a WGT event was experienced in *Brassica* ancestor approximately 22.5 MYA [17]. Furthermore, two model *Brassica* diploid species, *Brassica rapa* and *Brassica oleracea*, diverged from a common ancestor about 3.75 MYA [16, 18–20] (Fig. 1). The evolutionary history of angiosperms enriches the content of cytochrome P450 gene evolution group, which provides a clear evolutionary roadmap for the evolutionary history of cytochrome P450 genes. Aside from WGD or WGT, tandem duplication (TD) is another important mechanism that creates tandem duplicated genes. Tandem duplicated genes reside within segments of DNA that are repeated head-to-tail a number of times and are necessary for the maintenance of large gene families for expanding and contracting rapidly in response to demand of changing environment [21]. Tandem duplicated genes represent a large portion of the total genes in a genome, i.e., 14–17% of animal genomes [22]. A previous report revealed that tandem duplicated genes can rapidly change in response to the needs of the environment, causing an increase in genetic complexity [23]. These two types of evolutionary mechanisms play important roles in the evolution of cytochrome P450 supergene family.

To date, scholars have yet to elucidate the mechanism by which WGD and TD influence the expansion or reduction of cytochrome P450 gene families in the genus *Brassica*; they also have yet to identify which experienced stronger selection pressures of P450 orthologous gene pairs between or within *A. thaliana–B. rapa* and *A. thaliana–B. oleracea* lineages. The difference in expression patterns of cytochrome P450 gene families in *Brassica* species also remains undetermined. In this study, we identified the members of the cytochrome P450 gene superfamily in *B. rapa* and *B. oleracea* and compared them with those in *A. thaliana* on the basis of P450 gene superfamily size, genomic distribution, and phylogenetic analysis. The evolutionary history for the P450 gene super family and the contributions of WGD and TD to the evolution of different P450 gene families in *A. thaliana* and *Brassica* species were also investigated. We analyzed the expression patterns of different P450 gene families and the different types of P450s in different P450 gene families in *Brassica* species. According to the syntenic relationships between the *A. thaliana* and *Brassica* genomes, we detected the selection forces for inter- or intra-genome P450 gene pairs between or within *A. thaliana*–*B. rapa* and *A. thaliana*–*B. oleracea* lineages. Finally, we illustrated the evolution mechanisms of cytochrome P450 gene families in an attempt to uncover a clear evolutionary dynamic path that shaped this large and divergent gene superfamily accompanied with the evolutionary history of the genus *Brassica*.

## Methods

### Data resources

Genomic and annotation data for *A. thaliana*, *B. rapa*, and *B. oleracea* were retrieved from TAIR10 (http://www.arabidopsis.org) [24], BRAD (http://*Brassica*db.org/brad/) [25], and Bolbase (http://ocri-genomics.org/bolbase) [26], respectively. The putative datasets of tandem duplicated genes in *A. thaliana*, *B. rapa*, and *B. oleracea* were downloaded from PTGBase (http://ocri-genomics.org/PTGBase/) [27]. The hidden Markov model (HMM) profile of the cytochrome P450 domain (PF00067) was retrieved from Pfam 26.0 (http://pfam.xfam.org/) [28]. The RNA-seq data of *B. rapa* and *B. oleracea* were obtained from the Gene Expression Omnibus database with accession numbers GSE43245 and GSE42891, respectively [29]. The microarray data of *A. thaliana* were obtained from AtGenExpress resource [30].

### Sequence retrieval

Cytochrome P450 genes for the *A. thaliana* and *Brassica* genomes were identified through the HMM profile corresponding to the Pfam (p450) family PF00067 domain by using HMMER V3.0 [31], with the “trusted cutoff” as threshold. From the selected protein sequences filtered through the p450 domain, high-quality sequences between *A. thaliana* and *Brassica* species were aligned through ClustalW [32]. These sequences were then utilized to construct the species-specific HMM profile of cytochrome P450 by using the “hmmbuild” module of HMMER V3.0. With this species-specific HMM profile, all putative cytochrome P450 genes were identified in the *A. thaliana* and *Brassica* genomes. Finally, all cytochrome P450 genes in the *A. thaliana* and *Brassica* genomes were checked by using the conserved domain (Pfam: PF00067 and GENE3D: G3DSA:1.10.630.10) of cytochrome P450 genes.

### Physical map location

On the basis of the general feature format (GFF) file of the predicted genes in the *A. thaliana*, *B. rapa*, and *B. oleracea* genomes, 12 in-house Perl and Python scripts were used to process the data and draw the graphic portrayal of cytochrome P450s on chromosomes or pseudo-molecules with the SVG module.

### Alignments and phylogenetic analysis

For the phylogenetic analysis, the complete predicted protein sequences of cytochrome P450s that contain the full p450 domain in *A. thaliana*, *B. rapa*, and *B. oleracea* were aligned using MUSCLE with default options [33]. Using location of conserved P450 domains on P450 protein sequences, the alignment of the conserved domains of all cytochrome P450s among 3 species were processed through manual curation using Jalview [34]. The resulting alignment of conserved P450 domain of all P450s were used to construct a phylogenetic tree by using the maximum likelihood method with 1000 replications and the substitution model with the Jones–Taylor–Thornton model in MEGA 5.0 [35].

### Statistical tests

Significant difference in the ratios of the rates of non-synonymous to synonymous substitutions (Ka/Ks) of P450 orthologous gene pairs between two data samples was evaluated using the Mann–Whitney U test in the R programming language.

### RNA-seq data analysis of cytochrome P450s

For the expression values of cytochrome P450 genes, transcript abundance was calculated by fragments per kilobase of exon model per million mapped reads (FPKM) from CuffLinks v2.1.0, and the FPKM values were log2 transformed [36]. A hierarchical cluster was generated by using Cluster 3.0, and a heat map was displayed using TreeView 1.60 [37].

### Substitution rate estimates for P450s

The ratio of the rates of non-synonymous to synonymous substitutions (Ka/Ks) of all P450 gene pairs was evaluated for each branch of the phylogenetic tree by using PAML to estimate the selection mode for the P450 orthologous gene pairs in *A. thaliana* compared with *B. rapa* and *B. oleracea* [38]. For each subtree of P450 orthologous gene pairs between *A. thaliana*–*B. rapa* and *A. thaliana*–*B. oleracea* lineages, model 1 with Ka/Ks ratios was evaluated separately for the branches. The Ka/Ks values with terminal branches between modern species and their most recent reconstructed ancestors were used for the subsequent analyses.

## Results

### *Brassica* species has a larger P450 gene superfamily than *A. thaliana*

We identified the members of the P450 super gene family from the released protein sequences of *A. thaliana*, *B. rapa*, and *B. oleracea* to process an in-depth comparison between the cytochrome P450 gene family in *A. thaliana* and *Brassica* species. According to the conserved domains or motifs (Pfam: PF00067 and G3DSA: 1.10.630.10) of cytochrome P450 genes, we extracted 251 P450 genes from 27,416 *A. thaliana* representative protein-encoding genes based on TAIR10 genome release, which represented 0.916% of total representative protein-encoding genes in *A. thaliana*; these genes were distributed into 47 P450 gene families [28, 39]. Using consistent procedures, we identified 354 and 343 P450 genes from 41,174 *B. rapa* and 45,758 *B. oleracea* protein-encoding genes which represented 0.8598 and 0.7496% of total protein-encoding genes in *B. rapa* and *B. oleracea*, respectively. From comparison, P450s in *B. rapa* and *B. oleracea* were 1.41- and 1.37-fold as many as those in *A. thaliana* (Additional file 1: Table S1). After manually curation and combing the classification criteria of cytochrome P450 by previous studies, we classified the 948 P450 genes into 47 P450 gene families among the three species [4]. The Evolutionary analysis of P450s in different organisms revealed that P450 gene families are grouped into two classes: non-A type and A-type [7]. P450 gene families of non-A type share a sequence similarity to analogs in animal and fungi, but those of A-type are plant specific; the second type was generated after the divergence of plants from animals and fungi. The identified 948 P450s among the three species were classified into two classes. In *A. thaliana*, 94 and 157 P450s were clustered into non-A type and A-type, respectively. For *Brassica* species, 143 *B. rapa* genes and 136 *B. oleracea* genes were classified into non-A type and further divided into 28 families; the remaining 211 *B. rapa* and 207 *B. oleracea* genes were grouped into A-type P450s, which were divided into 19 families (Table 1). Comparison of A-type and non-A type P450s in *Brassica* species revealed the greater number of P450s in A-type than in non-A type, which suggested that plant-specific P450s underwent family member evolution unlike the ancestral P450s shared with animal and fungi in *Brassica* species. Due to classification of different clans of plant P450 families, 47 P450 gene families were classified into nine different clans of P450 gene families among *A. thaliana*, *B. rapa* and *B. oleracea*.

**Table 1:** Statistics of cytochrome P450 gene families in *A. thaliana* and *Brassica* species

Classifications		Family	*Arabidopsis thaliana*	*Brassica rapa*	*Brassica oleracea*
Different types	Different clans				
Non-A type	CYP51 clan	CYP51	1	3	5
	CYP72 clan	CYP714	2	1	2
		CYP715	1	4	3
		CYP709	3	6	7
		CYP72	9	18	6
		CYP721	1	1	1
		CYP734	1	1	1
		CYP735	2	3	2
	CYP710 clan	CYP710	4	3	2
	CYP711 clan	CYP711	1	1	1
	CYP74 clan	CYP74	2	2	2
	CYP85 clan	CYP702	6	4	3
		CYP707	4	8	9
		CYP708	4	6	3
		CYP716	3	0	0
		CYP718	1	1	1
		CYP720	1	1	1
		CYP722	1	1	2
		CYP724	1	2	2
		CYP85	2	4	4
		CYP87	1	2	2
		CYP88	2	2	2
		CYP90	4	9	9
	CYP86 clan	CYP704	3	5	4
		CYP86	11	13	14
		CYP94	6	10	10
		CYP96	14	30	32
	CYP97 clan	CYP97	3	2	6
Total non-A type			94	143	136
A-type	CYP71 clan	CYP701	1	1	1
		CYP703	1	2	2
		CYP705	25	22	3
		CYP706	8	10	9
		CYP71	50	73	77
		CYP712	2	2	2
		CYP73	1	6	5
		CYP75	1	1	1
		CYP76	9	12	12
		CYP77	5	5	5
		CYP78	6	11	12
		CYP79	10	11	11
		CYP81	18	32	37
		CYP82	5	4	9
		CYP83	2	6	6
		CYP84	2	4	6
		CYP89	7	4	3
		CYP93	1	1	2
		CYP98	3	4	4
Total A-type			157	211	207
Total			251	354	343

### Genomic distribution on chromosomes/pseudo-molecular chromosomes

According to the GFF file of cytochrome P450 genes between the *A. thaliana* and *Brassica* genomes, 251 (100%) P450s in *A. thaliana*, 337 (95.2%) in *B. rapa*, and 281 (81.92%) in *B. oleracea* were distributed on chromosomes or pseudo-molecule chromosomes, and the remaining 17 (4.8%) P450s in *B. rapa* and 62 (18%) P450s in *B. oleracea* were distributed on unanchored contigs or scaffolds [25, 26, 39]. For the distribution of P450s in the *A. thaliana* genome, Bak et al. finished this analysis based on previous analysis results of P450s [40]. In present study, Chr3 contained the greatest number of P450s (e.g., 68; 27.1% of the total P450s) among all the studied chromosomes, a consistent with Dr. Bak’s reports [40]. In the *B. rapa* genome, A03 (53; 15.63%) and A10 (16; 4.72%) contained the greatest and the least numbers of P450s, respectively. For the *B. oleracea* genome, C07 (55; 19.43%) and C05 (12; 4.24%) carried the greatest and the least numbers of P450s, respectively. The distribution of the members of P450 gene families on chromosomes or pseudo-molecules did not exhibit any of the same or similar regularity. For the members of the CYP72 gene family in the *A. thaliana* genome, 8 of 9 P450s were located on Chr2; the remaining 1 P450 was located on Chr1. For the members of the CYP72 gene family in the *B. rapa* genome, 18 P450s were located on six chromosomes (6 P450s on A05, 5 P450s on A03, 2 P450s in A01 and A06, 1 on each P450 on A08 and A09, and only 1 P450 on unanchored contigs). For the 5 members of the CYP72 gene family in the *B. oleracea* genome, 3 P450s were located on C08, 1 unanchored on chromosomes, and another was on C05 (Additional file 2: Figure S1).

### Phylogenetic analysis of cytochrome P450 genes in *B. rapa*, *B. oleracea*, and *A. thaliana*

On the basis of the phylogenetic analysis of cytochrome P450 genes among *A. thaliana*, *B. rapa*, and *B. oleracea*, 948 P450s were clustered into groups I and II (Additional file 3: Figure S2). Group I contained all non-A type P450 gene families, which were clustered into CYP51 clan, CYP710 clan, CYP711 clan, CYP72 clan, CYP74 clan, CYP85 clan, CYP86 clan, and CYP97 clan. Group II contained all A type P450 gene families, which were clustered into CYP71 clan. These results were consistent with previous study [8, 40]. Group I included 94, 143, and 134 P450s in *A. thaliana*, *B. rapa*, and *B. oleracea*, respectively, which covered 29 P450 gene families. Among the 29 P450 gene families, the CYP96 gene family (belonged to CYP86 clan) contained the most number of P450s in the three species, i.e., 14, 30, and 31 P450s in *A. thaliana*, *B. rapa*, and *B. oleracea*, respectively. The CYP716 gene family (belonged to CYP85 clan) had 3 P450s in *A. thaliana* based on TAIR10 but none in *B. rapa* and *B. oleracea*. This finding suggests that this family is lost in *B. rapa* and *B. oleracea*. There were two single specific family subgroups detected in group I; each of them contained one P450 gene in *B. oleracea*, which were Bol038708 and Bol016308, respectively. Analysis of protein sequences of these two P450s revealed these two P450s contained partial sequence of p450 conserved domain. Based on the basis of the classification criteria of P450 gene families, Bol038708 was classified into CYP82 gene family (belonged to CYP71 clan), and Bol016308 was classified into CYP96 gene family (belonged to CYP86 clan) in *B. oleracea*.

The A-type of P450 gene families (recognized as CYP71 clan) was classified into group II, which included 577 P450 genes among the three species. These P450 genes in II group were clustered into 23 P450 gene families, which represented 62.55% (157), 59.6% (211), and 60.93% (209) of the total P450s in *A. thaliana*, *B. rapa*, and *B. oleracea*, respectively. Of the 23 P450 gene families in group II, the CYP71 gene family, the largest A-type P450 gene family, contained 50, 73, and 77 P450 genes in *A. thaliana*, *B. rapa*, and *B. oleracea*, respectively. For the phylogenetic relationship of the members in the CYP71 gene family, members of the CYP83 and CYP71 gene families were clustered together. Members of the CYP83 gene family exhibited a closer evolutionary relationship than some members of the CYP71 gene family, indicating that members of the former showed closer relative relationship than some members of the latter. Except for clusters from 23 P450 gene families, one single specific family subgroup compared to *A. thaliana* was detected in the A-type P450 gene families, which contained 1 P450 gene (Bra014459) in *B. rapa*. Concerning Bra014459, sequence analysis revealed that it was formed by fragments of two different conserved domains: the first 125 amino acids correspond to a L-arabinokinase-like protein (“Glyco_trans_1–3 superfamily” domain), and the rest of the sequence correspond to a partial P450 protein. There was another single specific family subgroups existed in group II; this group contained 3 P450s (Bol042895, Bol009797, and Bol016015) in *B. oleracea*. These three P450s also contained partial sequences of p450 conserved domain of standard P450 protein through analysis of protein sequences. According to the basis of the classification criteria of P450 gene families, Bra014459 was classified into CYP76 gene family (belonged to CYP71 clan), and the three P450s (Bol042895, Bol009797, and Bol016015) from single specific family subgroup were classified into CYP709 (belonged to CYP72 clan), CYP72 (belonged to CYP72 clan), CYP96 (belonged to CYP86 clan) gene families in *B. oleracea* respectively.

### Synteny analysis of P450s between *A. thaliana* and *Brassica* species

*A. thaliana* and all the *Brassica*ceae taxa have experienced three rounds (i.e., γ, β, and α) of WGD or WGT in the genome evolutionary history of angiosperms. To trace the evolutionary history of the cytochrome P450 gene superfamily, we used the duplicated genes from three WGD or WGT events in *A. thaliana* [11]. After curation, we obtained 3530, 1421, and 505 syntenic gene pairs in the *A. thaliana* genome from the α, β, and γ WGD or WGT events, respectively. According to the new curated duplicated gene datasets based on TAIR10 genome release, only one P450 gene pair from the CYP94 gene family was retained after the γ WGT event, indicating that these two members of the CYP94 gene family existed before the γ WGD of eudicot ancestor. For the β WGD, we obtained three cytochrome P450 gene pairs distributed in the CYP86, CYP72, and CYP81 gene families, implying that these cytochrome P450 genes existed before the β WGD and retained the duplicated gene pairs after the β WGD. For duplicated gene pairs of the α WGD, we retrieved 22 P450 genes from the syntenic gene pairs caused by the α WGD, which were distributed in 11 P450 gene families. This result suggests that these 22 cytochrome P450 genes were retained after the most recent α duplication of *Brassica*ceae.

**Fig. 2:**
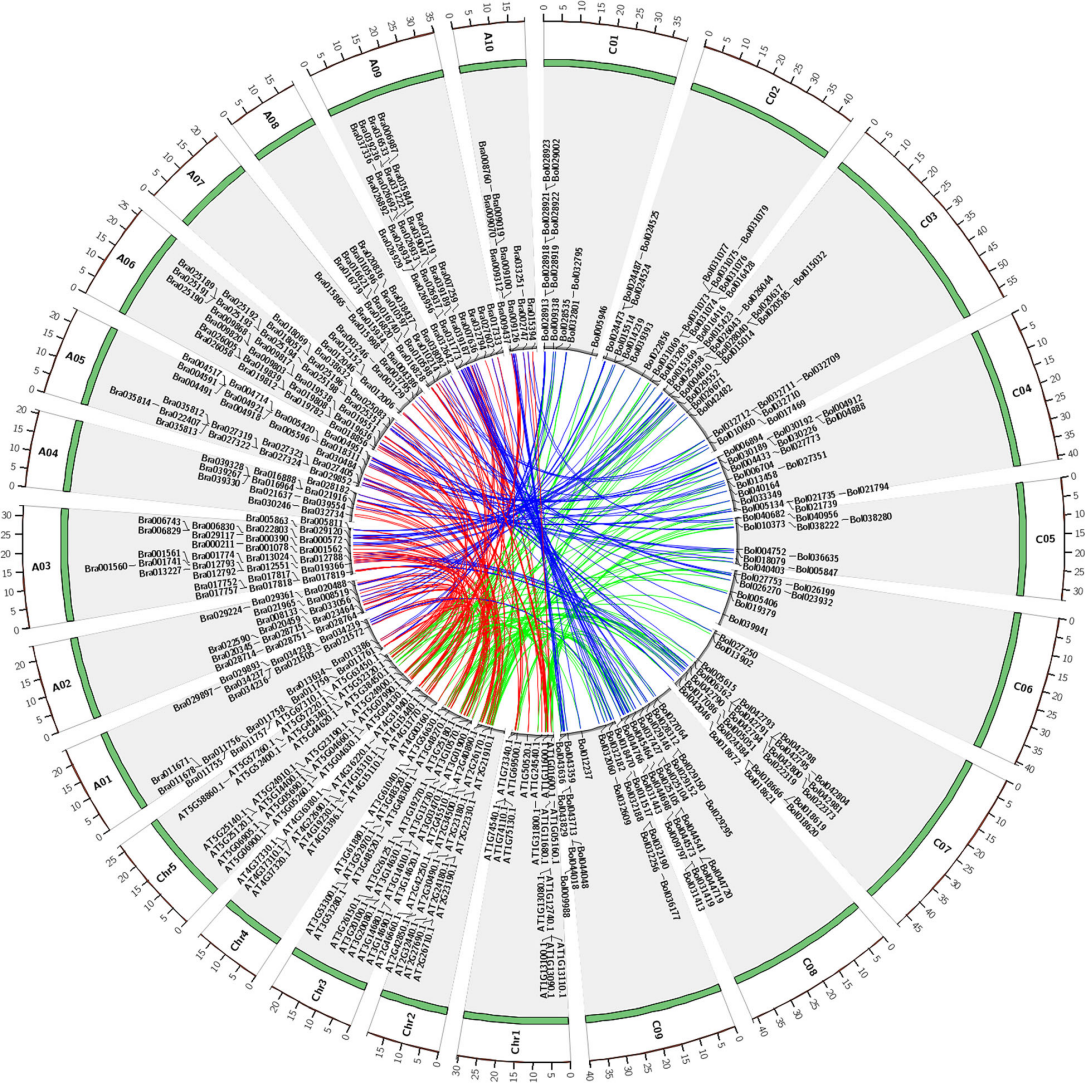
Syntenic relationships of cytochrome P450 genes between *A. thaliana* and *Brassica* species. Green bars represent pseudochromosomes of three species. A01–A10 represent pseudochromosomes in *B. rapa*, C01–C09 represent pseudochromosomes in *B. oleracea*, and Chr1–Chr5 represent pseudochromosomes in *A. thaliana*. Red lines represent the collinear relationships of P450 gene pairs between *A. thaliana* and *B. rapa*. Blue lines represent the collinear relationships of P450 gene pairs between *B. rapa* and *B. oleracea*. Green lines represent the collinear relationships of P450 gene pairs between *A. thaliana* and *B. oleracea*

After the split of *A. thaliana* and *Brassica* from a common ancestor, the *Brassica* ancestor experienced WGT and then diverged to yield the modern *B. rapa* and *B. oleracea*. The WGT brought triplicated genomic regions in the *B. rapa* and *B. oleracea* genomes. After the completion of the *B. rapa* and *B. oleracea* genome sequencing project, the triplicated regions were constructed in the *B. rapa* and *B. oleracea* genomes relating to the 24 ancestral crucifer blocks (A–X) in the *A. thaliana* genome [41, 42]. On the basis of the difference in gene density, the triplicated blocks in the two *Brassica* species were classified into three subgenomes: MF1 (medium fractionated), MF2 (most fractionated), and LF (least fractionated) [41]. According to the above-mentioned classification criteria of syntenic blocks, we reconstructed the triplicated regions in the *B. rapa* and *B. oleracea* genomes compared with that in the *A. thaliana* genome. On the basis of the syntenic relationships between *A. thaliana* and *Brassica* species, 131 *A. thaliana* P450s were found orthologous in *Brassica* species, which were distributed in 42 cytochrome P450 gene families. Then, 124 and 118 *A. thaliana* P450s were orthologous in 187 and 178 P450s in *B. rapa* and *B. oleracea*, respectively. From the syntenic regions in *B. rapa* and *B. oleracea* compared with *A. thaliana*, 162 orthologous gene pairs were identified between the *B. rapa* and *B. oleracea* genomes (Fig. 2). Of the 120 P450s in *A. thaliana*, 11 cytochrome P450s were detected in 3 orthologous genes and 41 P450s were detected in 2 orthologous genes in the *B. rapa* genome. The remaining 72 genes were detected in only one orthologous gene in the *B. rapa* genome. For 118 P450s in *A. thaliana* compared with the *B. oleracea* genome, 10, 39, and 69 *A. thaliana* P450s showed 3, 2, and 1 orthologous gene (s) in the *B. oleracea* genome, respectively (Additional file 4: Table S2).

According to the syntenic relationship between *A. thaliana* and *Brassica* species, the duplicated gene pairs from different polyploidy events in *A. thaliana* were orthologous in different subgenomes in *Brassica* species. One duplicated gene pair of the CYP94 gene family (AT2G27690.1 and AT3G56630.1) from the γ WGT was detected in 6 corresponding orthologous genes in *Brassica* species. AT2G27690.1 was detected in 1 orthologous gene and AT3G56630.1 was detected in 2 orthologous genes in different subgenomes in *B. rapa* or *B. oleracea*. For the β WGD, three duplicated gene pairs were detected in 21 orthologous P450 genes in *Brassica* species. For example, one member (AT1G17060.1) of the duplicated gene pairs (AT1G17060.1 and AT3G14690.1) from the CYP72 family was detected in 3 orthologous P450 genes in *B. rapa* and *B. oleracea*, but another P450 gene (AT3G14690.1) was detected in 3 orthologous P450 genes in *B. rapa* and 1 orthologous P450 gene in *B. oleracea*. This result indicates that these two *A. thaliana* P450s showed significant expansion in the genus *Brassica*. The 11 duplicated gene pairs from the α WGD were also detected in 63 orthologous P450 genes in *Brassica* species, and each duplicated gene has its corresponding P450s in *B. rapa* or *B. oleracea*. Of the 22 duplicated genes in *A. thaliana*, only AT4G37370.1 and AT5G45340.1 from the CYP81 and CYP707 gene families showed 3 copies that were retained in *B. rapa* and *B. oleracea*, respectively; other *A. thaliana* P450s showed 1 or 2 copies that were retained in *Brassica* species (Table 2).

**Table 2:** Retention or loss of P450 orthologous gene pairs between *A. thaliana* and *Brassica* species from different polyploidy events

Polyploidy events	*Brassica rapa*			*Brassica oleracea*			Gene	Gene name	Family	Orthologous genes	Gene name	*Brassica rapa*			*Brassica oleracea*		
	BraLF	BraMF1	BraMF2	BolLF	BolMF1	BolMF2						BraLF	BraMF1	BraMF2	BolLF	BolMF1	BolMF2
α	Bra033251	Bra032642	NA	Bol040682	Bol018470	NA	AT1G01600.1	CYP86A4	CYP86	AT4G00360.1	CYP86A2	Bra037336	NA	Bra008519	Bol011517	NA	Bol040623
	Bra015394	NA	NA	Bol040956	NA	NA	AT1G05160.1	CYP88A3	CYP88	AT2G32440.1	CYP88A4	Bra005596	NA	NA	Bol013458	NA	NA
	Bra018856	NA	Bra030484	NA	NA	NA	AT1G50520.1	CYP705A27	CYP705	AT3G20130.1	CYP705A22	Bra035812	NA	NA	Bol004752	NA	NA
	NA	Bra034021	NA	NA	NA	NA	AT1G67110.1	CYP735A2	CYP735	AT5G38450.1	CYP735A1	NA	NA	NA	Bol044541	NA	NA
	NA	Bra031222	NA	NA	Bol044766	NA	AT2G21910.1	CYP96A5	CYP96	AT4G39490.1	CYP96A10	Bra007636	NA	NA	Bol044573	Bol037477	NA
	Bra030246	NA	NA	Bol031784	NA	NA	AT2G22330.1	CYP79B3	CYP79	AT4G39950.1	CYP79B2	Bra037336	NA	Bra008519	Bol011517	NA	Bol040623
	NA	Bra039189	Bra000572	NA	Bol044719	Bol015032	AT2G23190.1	CYP81D7	CYP81	AT4G37370.1	CYP81D8	Bra013634	Bra019366	Bra020836	Bol028535	Bol042046	Bol010650
	NA	Bra039330	NA	NA	Bol021735	NA	AT2G45550.1	CYP76C4	CYP76	AT3G61040.1	CYP76C7	NA	NA	Bra010214	NA	NA	Bol017469
	Bra004517	Bra039267	NA	Bol004912	Bol021794	NA	AT2G46660.1	CYP78A6	CYP78	AT3G61880.1	CYP78A9	NA	NA	Bra005811	NA	NA	Bol015369
	Bra029852	NA	NA	Bol005847	NA	NA	AT3G10570.1	CYP77A6	CYP77	AT5G04660.1	CYP77A4	Bra028182	NA	NA	Bol040164	NA	NA
	Bra013386	Bra012551	NA	Bol009338	Bol024384	NA	AT4G19230.1	CYP707A1	CYP707	AT5G45340.1	CYP707A3	Bra025083	Bra027602	Bra021965	Bol005615	Bol032060	Bol022856
β	Bra026005	Bra026692	Bra016621	Bol038280	Bol009797	Bol029295	AT1G17060.1	CYP72C1	CYP72	AT3G14690.1	CYP72A15	Bra027319	Bra021572	Bra001562	Bol002804	NA	NA
	Bra011755	NA	NA	Bol028923	NA	NA	AT4G37310.1	CYP81H1	CYP81	AT5G67310.1	CYP81G1	Bra012152	Bra037119	NA	Bol027250	NA	NA
	Bra033251	Bra032642	NA	Bol040682	Bol018470	NA	AT1G01600.1	CYP86A4	CYP86	AT2G45970.1	CYP86A8	Bra004951	NA	NA	Bol030226	NA	NA
γ	NA	Bra012006	NA	NA	Bol013448	NA	AT2G27690.1	CYP94C1	CYP94	AT3G56630.1	CYP94D2	Bra007259	NA	Bra003246	Bol045464	NA	Bol004209

### Contributions of tandem duplication to family size

Gene families organized in tandem arrays of two or more units have been described in *A. thaliana*, *B. rapa*, and *B. oleracea* [41–43]. The putative tandem duplicated genes of cytochrome P450 in *A. thaliana, B. rapa*, and *B. oleracea* were extracted from PTGBase (http://ocri-genomics.org/PTGBase/) [27]. According to the classification criteria of cytochrome P450 gene families, we curated the putative datasets of tandem duplicated genes among the three species. We obtained 128, 137, and 112 tandem duplicated P450 genes in *A. thaliana*, *B. rapa*, and *B. oleracea*, respectively (Additional file 5: Table S3). Analysis of P450s in the *A. thaliana* genome revealed that 128 P450s, representing 51% of the total P450s in *A. thaliana*, were generated by TD and were distributed in 40 tandem arrays with 2–9 P450s. Among the 128 cytochrome P450s in the *A. thaliana* genome, 39 P450s were members of the CYP71 gene family, representing 30.47% of the total tandem duplicated genes of cytochrome P450s in the *A. thaliana* genome. This result indicates that the CYP71 gene family was influenced more strongly by the TD event compared with the other P450 gene families in *A. thaliana*. Among the 354 *B. rapa* P450 genes, 137 (38.70%) were generated by TD and were distributed in 47 tandem arrays with 2–10 P450s. All tandem duplicated P450s in the *B. rapa* genome were distributed in 18 gene families. The CYP71 gene family in *B. rapa* contained 44 tandem duplicated P450s, representing 12.36% of the total tandem duplicated P450s, which contained most of the tandem duplicated P450s compared with the other P450 gene families in *B. rapa*. In the *B. oleracea* genome, 112 of the total 343 identified P450 genes, representing 32.65% of the total *B. oleracea* cytochrome P450s, were detected in 40 tandem arrays of 2–6 genes. Similarly, the CYP71 gene family also contained more tandem duplicated P450 genes compared with other P450 families in *B. oleracea*. These results suggest that the P450 genes in *A. thaliana* were influenced more strongly by the TD event compared with the other genes in *Brassica* species and that the CYP71 gene family was influenced more strongly by the TD event compared with the other gene families among the three species (Table 3).

**Table 3:** Tandem duplicated genes of cytochrome P450 in *A. thaliana*, *B. rapa*, and *B. oleracea*

Categories	*Arabidopsis thaliana*	*Brassica rapa*	*Brassica oleracea*
CYP702	4	2	0
CYP704	0	2	0
CYP705	19	7	0
CYP706	6	2	2
CYP708	0	2	0
CYP709	2	4	5
CYP71	39	44	40
CYP710	4	2	0
CYP714	2	0	0
CYP715	0	2	2
CYP716	2	0	0
CYP72	8	13	4
CYP73	0	4	4
CYP76	8	7	7
CYP77	4	0	0
CYP78	0	2	0
CYP79	6	0	0
CYP81	10	20	23
CYP82	3	0	2
CYP83	0	2	2
CYP84	0	0	2
CYP86	2	0	0
CYP89	3	2	0
CYP94	0	2	2
CYP96	4	18	17
CYP98	2	0	0
Total tandem duplicated genes	128	137	112
Total P450 genes	251	354	343
Percentage	51%	38.7%	32.65%

We traced the ancient tandem duplicated genes of cytochrome P450 before the split of *A. thaliana* and its *Brassica* ancestor to investigate the effects of WGD on the tandem duplicated P450s. Basing from the syntenic relationships among the *B. rapa*, *B. oleracea*, and *A. thaliana* genomes, we used syntenic gene pairs among the three species to classify the ancient units of tandem duplicated genes. After the curation of orthologous gene pairs among the three species, we detected that 24 *A. thaliana* P450s, distributed in tandem arrays of 2–6 P450 genes, obtained 28 and 17 corresponding orthologous genes in the *B. rapa* and *B. oleracea* genomes, respectively, which were located on different subgenomes in the *Brassica* genomes. Among the 24 *A. thaliana* P450s, 12 race specific P450s co-retained the corresponding orthologous P450 genes in the *Brassica* genomes after the TD event, and 12 lineage-specific P450s co-retained 1 corresponding orthologous P450 gene in the *B. rapa* or *B. oleracea* genome. These ancient units of tandem duplicated genes have existed before the split of *A. thaliana* and its *Brassica* ancestor and have been retained after the divergence of *B. rapa* and *B. oleracea*. For 128 P450s in *A. thaliana*, 24 P450s existed before the divergence of *A. thaliana* and its *Brassica* ancestor, and 104 P450s were species-specific tandem duplicated genes in the *A. thaliana* genome. This result indicates that cytochrome P450 gene families experienced species-specific expansion by TD after species divergence. A total of 109 and 95 P450s in the *B. rapa* and *B. oleracea* genomes, respectively, were found to be species specific after excluding the ancient tandem duplicated P450s from the two species. The tandem duplicated P450 genes in the *B. rapa* and *B. oleracea* genomes were composed of two parts. One part contained the ancient P450s generated before the split of *A. thaliana* from its *Brassica* ancestor; the other part contained the recent P450 members generated after the divergence of *B. rapa* and *B. oleracea* from a common ancestor (Table 4).

**Table 4:** Ancient tandem arrays of cytochrome P450 genes in *A. thaliana* compared with *B. rapa* and *B. oleracea*

Block	AGI	Gene name	Family	Location	*Brassica rapa*			*Brassica oleracea*		
					BraLF	BraMF1	BraMF2	BolLF	BolMF1	BolMF2
A	AT1G13080.1	CYP71B2	CYP71	Chr1	/	/	/	/	Bol031411	/
A	AT1G13090.1	CYP71B28	CYP71	Chr1	/	Bra026934	/	/	Bol031412	/
A	AT1G13100.1	CYP71B29	CYP71	Chr1	/	Bra026933	/	/	Bol031413	/
F	AT3G14610.1	CYP72A7	CYP72	Chr3	Bra027324	/	/	/	/	/
F	AT3G14620.1	CYP72A8	CYP72	Chr3	Bra027323	/	/	/	/	/
F	AT3G14630.1	CYP72A9	CYP72	Chr3	Bra027322	/	/	/	/	/
F	AT3G14680.1	CYP72A14	CYP72	Chr3	/	/	Bra001561	/	/	/
F	AT3G14690.1	CYP72A15	CYP72	Chr3	/	/	Bra001562	/	/	/
F	AT3G20130.1	CYP705A22	CYP705	Chr3	Bra035812	/	/	/	/	/
F	AT3G20140.1	CYP705A23	CYP705	Chr3	Bra035813	/	/	/	/	/
L	AT3G26170.1	CYP71B19	CYP71	Chr3	Bra025189	/	/	Bol042791	/	/
L	AT3G26180.1	CYP71B20	CYP71	Chr3	Bra025190	/	/	Bol042790	/	/
L	AT3G26190.1	CYP71B21	CYP71	Chr3	Bra025191	/	/	Bol042793	/	/
L	AT3G26200.1	CYP71B22	CYP71	Chr3	Bra025192	Bra036345	/	Bol042794	Bol031073	/
L	AT3G26210.1	CYP71B23	CYP71	Chr3	Bra025193	Bra036344	/	Bol042795	Bol031074	/
L	AT3G26220.1	CYP71B3	CYP71	Chr3	Bra025194	Bra036343	/	/	Bol031075	/
L	AT3G26280.1	CYP71B4	CYP71	Chr3	/	/	Bra034236	/	/	/
L	AT3G26290.1	CYP71B26	CYP71	Chr3	/	/	Bra034237	/	/	/
L	AT3G26320.1	CYP71B36	CYP71	Chr3	/	/	Bra034238	/	/	/
L	AT3G26330.1	CYP71B37	CYP71	Chr3	/	/	Bra034239	/	/	/
U	AT4G37320.1	CYP81D5	CYP81	Chr4	/	Bra017817	/	/	Bol018621	/
U	AT4G37330.1	CYP81D4	CYP81	Chr4	/	Bra017818	/	/	Bol018620	/
U	AT4G37400.1	CYP81F3	CYP81	Chr4	Bra011758	/	Bra010597	Bol028919	/	Bol032711
U	AT4G37410.1	CYP81F4	CYP81	Chr4	Bra011759	/	Bra010598	Bol028918	/	Bol032712

### Influence of WGD and TD events on the P450 gene superfamily

After the divergence of *A. thaliana* and *Brassica* lineage from a common ancestor, cytochrome P450 gene families experienced different evolutionary patterns under environmental selection pressure. The WGD and TD events played important roles in the evolution of cytochrome P450 gene families. A previous study revealed that all *A. thaliana* orthologous genes show a 1.2-fold retention rate in syntenic regions in *Brassica* species through syntenic analysis between the *A. thaliana* and *Brassica* genomes [42]. Comparison of the retention rates of P450 orthologous genes in *A. thaliana* with *Brassica* genomes revealed that 1.5-fold *A. thaliana* P450s were retained in different subgenomes in *Brassica* species, which was higher than the retention rate of all *A. thaliana* orthologous genes in syntenic regions in *Brassica* species. This result suggests that WGD or WGT events caused significant increase of the members of P450 gene families in *Brassica* species. In the present study, we compared the influences of WGD and TD events on P450 gene families. Comparison of the influences of WGD events on P450s in *Brassica* species demonstrated that 124 and 117 *A. thaliana* P450s existed in 187 and 177 orthologous P450 genes in *B. rapa* and *B. oleracea*, respectively. This result reflects an identical retention rate of 1.5-fold of *A. thaliana* orthologous genes in *B. rapa* and *B. oleracea*. These P450s influenced by WGD represented 52.82 and 51.6% of the total P450s in *B. rapa* and *B. oleracea*, respectively*.* Analysis of influences of TD on P450s among the three species revealed that 128, 137, and 112 P450s in *A. thaliana*, *B. rapa*, and *B. oleracea* were generated by TD, representing 51% (251), 38.7% (354), and 32.65% (343) of the total P450s in the three species, respectively. These results indicated that WGD caused more influence on the evolution of P450 gene families than TD in the *Brassica* lineage and that TD exerted more influence on the evolution of P450 gene families in *A. thaliana* than in *Brassica* species.

For each P450 gene family, we summarized the members of P450 gene families that were generated by WGT or TD events based on the two classes: non-A type and A-type. Of the 28 non A-type P450 gene families, 22 (8 families in *A. thaliana*, 9 families in *B. rapa*, and 5 families in *B. oleracea*) were influenced by TD, but 23 and 24 P450 gene families were influenced by WGD in *B. rapa* and *B. oleracea*, respectively. Of the 19 A-type P450 gene families, 10, 9, and 8 P450 gene families were influenced by TD in *A. thaliana*, *B. rapa*, and *B. oleracea*, respectively, but 18 P450 gene families were influenced by WGD in each *B. rapa* and *B. oleracea*. Of the 137 and 112 tandem duplicated P450 genes in *B. rapa* and *B. oleracea*, 90 and 82 were distributed in A-type P450 supergene families, which represented 65.69 and 73.21% of the total tandem duplicated P450 genes in *Brassica* species, respectively. Among the 187 and 177 P450s from WGD in *B. rapa* and *B. oleracea*, 57.75 and 59.89% were distributed in A-type P450 supergene families. These results indicate that A-type P450 gene families were influenced more strongly by TD and WGD compared with non-A type P450 gene families in the *Brassica* lineage (Table 5).

**Table 5:** Comparison of cytochrome P450 genes in different types among *A. thaliana*, *B. rapa*, and *B. oleracea*

Classification		Family	*Arabidopsis thaliana*			*Brassica rapa*			*Brassica oleracea*		
Different types	Different clans		Total P450s	P450s from WGD	P450s from TD	Total P450s	P450s from WGD	P450s from TD	Total P450s	P450s from WGD	P450s from TD
Non-A Type	CYP51 clan	CYP51	1	1	0	3	2	0	5	2	0
	CYP72 clan	CYP714	2	2	2	1	1	0	2	2	0
		CYP715	1	1	0	4	2	2	3	2	2
		CYP709	3	1	2	6	1	4	7	1	5
		CYP72	9	6	8	18	11	13	6	5	4
		CYP721	1	1	0	1	1	0	1	1	0
		CYP734	1	1	0	1	1	0	1	1	0
		CYP735	2	2	0	3	2	0	2	1	0
	CYP710 clan	CYP710	4	1	4	3	2	2	2	1	0
	CYP711 clan	CYP711	1	1	0	1	0	0	1	1	0
	CYP74 clan	CYP74	2	1	0	2	1	0	2	1	0
	CYP85 clan	CYP702	6	2	4	4	3	2	3	1	0
		CYP707	4	3	0	8	7	0	9	7	0
		CYP708	4	0	0	6	0	2	3	0	0
		CYP716	3	0	2	0	0	0	0	0	0
		CYP718	1	1	0	1	1	0	1	1	0
		CYP720	1	1	0	1	1	0	1	1	0
		CYP722	1	0	0	1	0	0	2	0	0
		CYP724	1	1	0	2	2	0	2	2	0
		CYP85	2	0	0	4	0	0	4	0	0
		CYP87	1	1	0	2	2	0	2	2	0
		CYP88	2	2	0	2	2	0	2	2	0
		CYP90	4	3	0	9	7	0	9	7	0
	CYP86 clan	CYP704	3	2	0	5	3	2	4	3	0
		CYP86	11	9	2	13	13	0	14	13	0
		CYP94	6	5	0	10	9	2	10	9	2
		CYP96	14	3	4	30	3	18	32	2	17
	CYP97 clan	CYP97	3	2	0	2	2	0	6	3	0
Total non-A type			94	53	28	143	79	47	136	71	30
A Type	CYP71 clan	CYP701	1	1	0	1	1	0	1	1	0
		CYP703	1	0	0	2	0	0	2	0	0
		CYP705	25	5	19	22	6	7	3	2	0
		CYP706	8	2	6	10	3	2	9	4	2
		CYP71	50	28	39	73	37	44	77	35	40
		CYP712	2	2	0	2	2	0	2	2	0
		CYP73	1	1	0	6	3	4	5	3	4
		CYP75	1	1	0	1	1	0	1	1	0
		CYP76	9	5	8	12	4	7	12	5	7
		CYP77	5	4	4	5	5	0	5	5	0
		CYP78	6	5	0	11	8	2	12	9	0
		CYP79	10	3	6	11	4	0	11	4	0
		CYP81	18	12	10	32	23	20	37	23	23
		CYP82	5	2	3	4	2	0	9	2	2
		CYP83	2	1	0	6	1	2	6	1	2
		CYP84	2	2	0	4	2	0	6	3	2
		CYP89	7	1	3	4	1	2	3	1	0
		CYP93	1	1	0	1	1	0	2	1	0
		CYP98	3	2	2	4	4	0	4	4	0
Total A-type			157	78	100	211	108	90	207	106	82
Total			251	131 (52.19%)	128 (51%)	354	187 (52.82%)	137 (38.7%)	343	177 (51.6%)	112 (32.65%)

### Family-specific evolution for P450 gene families

**Fig. 3:**
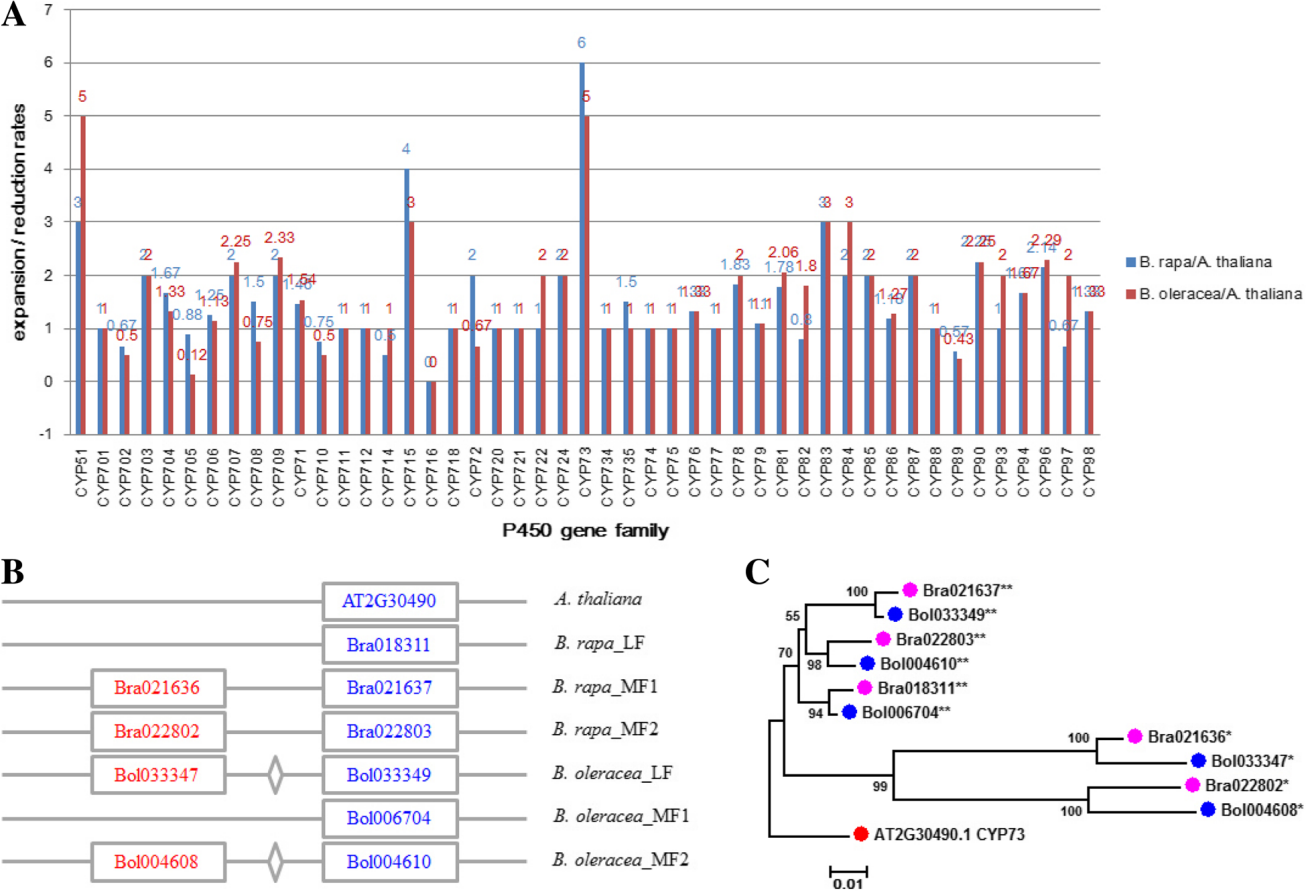
Comparison of expansion/reduction rates of P450 gene families and characteristics of CYP73 gene family members between *A. thaliana* and *Brassica* species. **a** Comparison of expansion/reduction rates of P450 gene families between *A. thaliana* and *Brassica* species. **b** Distribution of the member (s) of the CYP73 gene family in the *A. thaliana* and *Brassica* genomes. Gray lines represent the genomic or subgenomic regions among the *A. thaliana*, *B. rapa*, and *B. oleracea* genomes. Blue rectangles represent P450 genes, and diamonds represent non-P450 genes. **c** Phylogenetic relationship of CYP73 gene family members among *A. thaliana*, *B. rapa*, and *B. oleracea*. Red, pink, and blue solid circles represent P450 genes in *A. thaliana*, *B. rapa*, and *B. oleracea*, respectively. P450 genes with two stars represent P450s from whole genome duplication, and P450 genes with one star represent P450s from tandem duplication

WGD and TD triggered the evolution of P450 gene families in *B. rapa* and *B. oleracea* compared with *A. thaliana.* We defined the expansion/reduction rate to measure the expansion or reduction of the members of P450 gene families in *B. rapa* and *B. oleracea* compared with *A. thaliana*. The expansion/reduction rate is the ratio between the number of members of P450 gene families in *B. rapa* or *B. oleracea* and the number of members of corresponding P450 gene families in *A. thaliana.* An expansion/reduction rate greater than 1, less than 1, or equal to 1 represents expansion, reduction, or no difference in P450 gene families in *B. rapa* and *B. oleracea* compared with *A. thaliana*, respectively. According to the definition of the expansion/reduction rate of P450 gene families, 26, 13, and 8 P450 gene families showed expansion, no difference, and reduction of P450 gene families in *B. rapa* compared with *A. thaliana*, respectively*.* CYP73 gene families had higher expansion/reduction rate and showed more significant expansion than other families in *B. rapa* compared with *A. thaliana.* A total of 27, 13, and 7 P450 gene families indicated expansion, no difference, and reduction in *B. oleracea* compared with *A. thaliana*, respectively. CYP51 and CYP73 gene families had higher expansion/reduction rate and showed more significant expansion than other families in *B. oleracea* compared with *A. thaliana* (Fig. 3a).

We used the CYP73 gene family, which showed the highest expansion/reduction rate among the P450 gene families in *Brassica* species, as an example to analyze further the expansion of P450 gene families. The CYP73 gene family only have 1 P450s (AT2G30490.1, named CYP73A5) in *A. thaliana*. This P450 gene can encode a cinnamate-4-hydroxylase, and mutations in this gene affect phenylpropanoid metabolism, growth, and development [44]. In the *Brassica* lineage, we obtained five corresponding orthologous P450 genes in each *B. rapa* and *B. oleracea* compared with P450s in *A. thaliana.* Analysis of WGD revealed that the P450 gene (AT2G30490.1) in *A. thaliana* obtained three orthologous genes retained in triplicated blocks in *B. rapa* and *B. oleracea*. Of the three orthologous genes (Bra018311, Bra021637, and Bra022803 located on BraLF, BraMF1, and BraMF2, respectively) of the *A. thaliana* P450 gene (AT2G30490.1) in *B. rapa*, two (Bra021637 and Bra022803 located on BraMF1 and BraMF2) underwent TD and generated two paralogous genes (Bra021636 and Bra022802) in *B. rapa*. In *B. oleracea*, two P450s (Bol033349 and Bol004610 located on BolLF and BolMF2) experienced TD and generated two paralogous genes (Bol033347 and Bol004608) among triplicated genes (Bol033349, Bol006704, and Bol004610 located on BolLF, BolMF1, and BolMF2, respectively) (Fig. 3b). Phylogenetic analysis revealed that the triplicated P450 genes from *Brassica* species were clustered into one subgroup, and tandem duplicated P450 genes from triplicated P450 genes were clustered into the other subgroup, indicating closer relative relationships among triplicated P450 genes in the *Brassica* lineage (Fig. 3c). These results suggest that the *A. thaliana* orthologous P450 gene (AT2G30490.1) underwent the same evolutionary paths and that the WGD and TD evolutionary events were both responsible for the expansion of *A. thaliana* P450 gene (AT2G30490.1) in the *Brassica* lineage.

### Expression analysis of the members of cytochrome P450 gene families in *A. thaliana* and the *Brassica* lineage

**Fig. 4:**
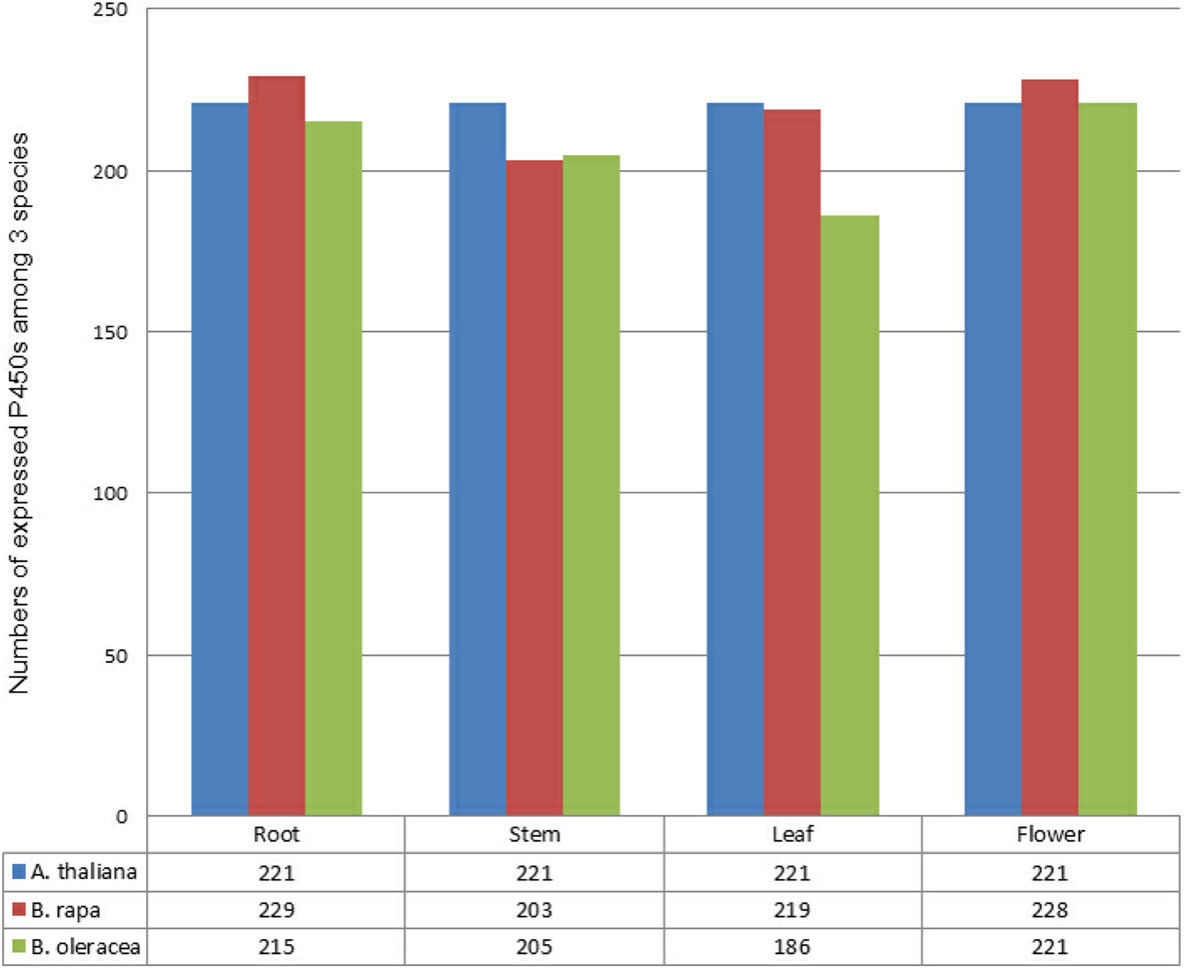
Comparison of the numbers of expressed P450s in different tissues in *A. thaliana* and *Brassica species*

Using microarray data from the developmental set of *A. thaliana*, we selected four tissues (root with 21 days; stem, 2nd internode with 21+ days; cauline leaves with 21+ days and flowers stage 9 with 21+ days) for expression analysis of 251 P450s in *A. thaliana* [30]. After curation, 221 P450s in *A. thaliana* were expressed in the four tissues. According to the expression profiling analysis from the FPKM, 354 and 343 P450 genes in *B. rapa* and *B. oleracea* were used to investigate the expression patterns of P450s in four tissues (root, stem, leaf and flower) in the genus *Brassica*. In total, 296 and 278 P450s were expressed in the four tissues in *B. rapa* and *B. oleracea*, respectively (Additional file 6: Table S4). The expression distribution of these genes among *A. thaliana*, *B. rapa* and *B. oleracea* in the four tissues is detailed by the histogram (Fig. 4). From microarray data analysis of all *A. thaliana* P450s, 221 P450s were all expressed in the four tissues in *A. thaliana* although some P450s showed very low expression values in certain tissues. But for the RNA-seq analysis of P450s in *Brassica* lineage, some P450s indicated tissue-specific expression in certain tissues in *B. rapa* or *B. oleracea.* In leaf and stem tissues of *Brassica* species, relatively few expressed P450s were identified compared with other tissues in *B. rapa* and *B. oleracea*, indicating less P450s participating in the biosynthesis of various primary and secondary metabolites in these two tissues. The number of P450s expressed in the leaf tissue in *B. rapa* was higher than that in *B. oleracea*, suggesting relatively more P450s were expressed in the tissue in *B. rapa*. For the comparative analysis of expressed P450 gene family, all members of 20 P450s gene family were expressed in one or more tissues, and most of the members of 26 P450s gene family were expressed in one or more tissues among *A. thaliana* and *Brassica* species. For the CYP703 gene family, there was only one P450s identified in *A. thaliana* and this P450s (AT1G01280.1, named CYP703A2) was expressed in the four tissues in *A. thaliana*. There were 2 P450s identified in *B. rapa* and *B. oleracea* respectively, but no P450s was detected to express in *B. rapa*; only one P450s was detected to express in *B. oleracea*. For the CYP716 gene family, 2 of 3 P450s were detected to express in four tissues in *A. thaliana*, although the member of CYP716 gene family was not identified in *Brassica* species (Additional file 7: Table S5)*.*

### Expression difference of P450s of different types in P450 gene families in *A. thaliana* and *Brassica* lineage

**Fig. 5:**
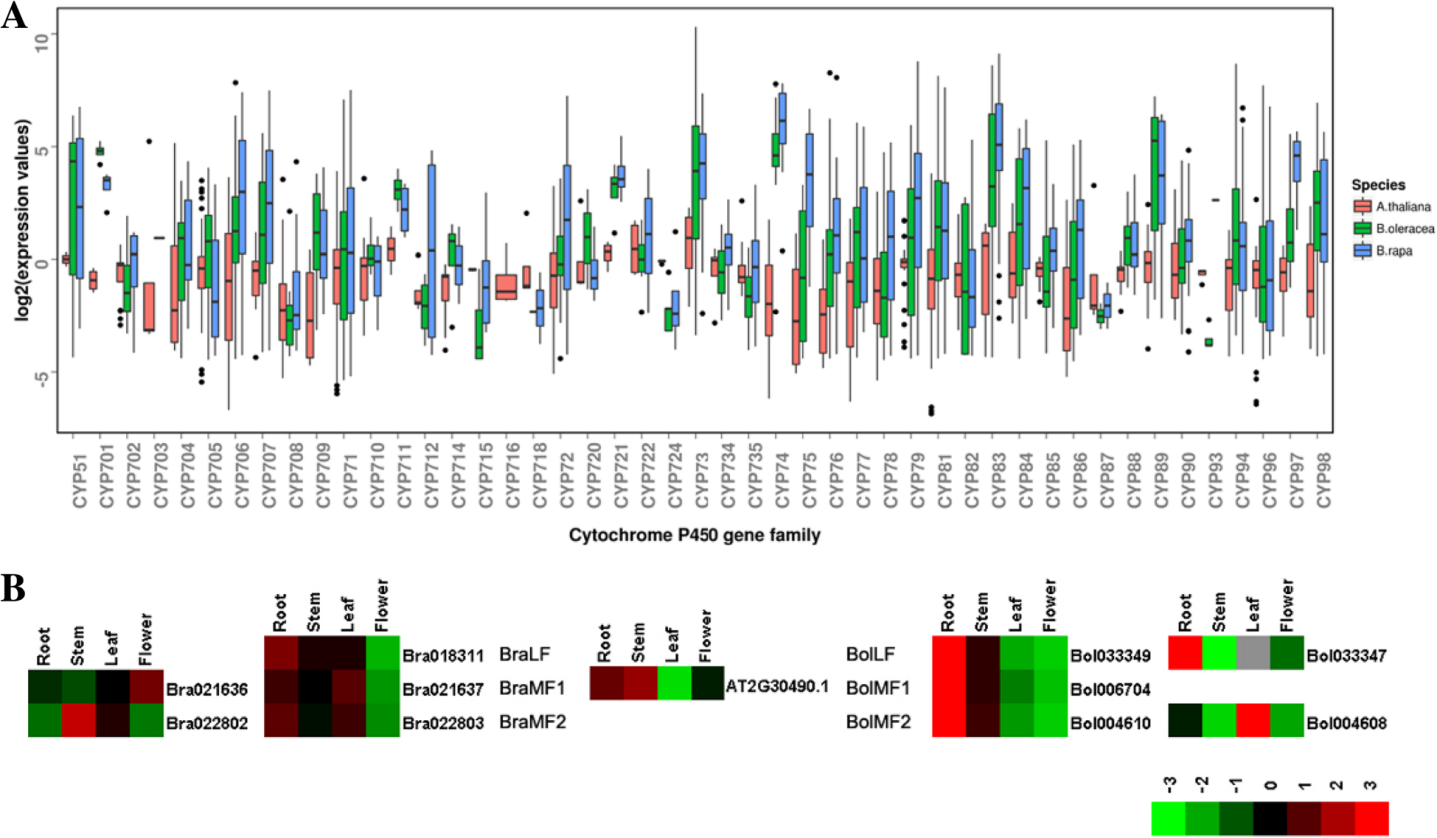
Boxplot comparisons of expression of P450s in different gene families and expression analysis of CYP73 gene family members in *A. thaliana* and *Brassica* species. **a** Boxplot comparisons of expression of P450s in different gene families in *A. thaliana*, *B. rapa* and *B. oleracea*. **b** Expression analysis of CYP73 gene family members across different tissues in *A. thaliana*, *B. rapa* and *B. oleracea*

The discrete degree of expression level of P450s can reflect the functional divergence of different P450 genes, which can be used to investigate the difference in expression pattern among the different P450 gene families in *A. thaliana* and *Brassica* species. For the expression analysis of P450s in the four tissues in *A. thaliana* and *Brassica* species, we employed quartile map to display the expression difference of various P450 gene families (Fig. 5a). Results showed that the expression values of the members of CYP73 gene family in *A. thaliana* indicated more discrete than those in other P450 gene families. In *Brassica* lineage, the expression values of CYP73 genes in *B. rapa* or *B. oleracea* were more discrete than those of genes from most of P450 families in *A. thaliana*. For the members of CYP73 gene family, the expression values of P450s in *B. oleracea* were more discrete than those in *B. rapa*, but the difference was not significant (*P* = 0.745 < 0.05, Mann-Whitney U test). Analysis of expansion of the CYP73 gene family uncovered that WGD and TD were both responsible for the expansion of the *A. thaliana* P450 gene (AT2G30490.1) in the *Brassica* lineage. After curation of the members of P450 gene families that underwent WGD or TD, we classified P450 genes in *Brassica* species into two types: P450s generated by WGD (WGD-type) and P450s generated by TD (TD-type). Therefore, five corresponding orthologous P450 genes in *B. rapa* or *B. oleracea* were divided into two TD-type P450s and three WGD-type P450s in *Brassica* species.

To investigate functional divergence, we compared the expression difference of the members of CYP73 gene family between *A. thaliana* and *Brassica* species, as well as intra-genome different types of the P450s from CYP73 gene family in *Brassica* species. In *A. thaliana*, the *A. thaliana* P450 gene (AT2G30490.1) was highly expressed in the stem tissue but lowly expressed in the leaf tissue. For the P450s in *Brassica* lineage, the two different types of P450s from CYP73 gene family indicated different expression patterns in *B. rapa* and *B. oleracea*. In *B. rapa*, the expression levels of WGD-type P450s (Bra018311, Bra021637, and Bra022803) were nearly the same across different tissues. These P450s were highly expressed in the root and leaf but lowly expressed in the flower. The TD-type P450s (Bra021636 and Bra022802) displayed different expression patterns compared with their other member of two-copy tandem array in the WGD-type P450s. For example, Bra021636 and Bra021637 were two-copy tandem duplicated genes located on BraMF1 regions in *B. rapa* genome. Bra021636 showed high expression in the flower and low expression in the stem, but Bra021637 showed high expression in the leaf and low expression in the flower. Bra022802 and Bra022803 were also two-copy tandem duplicated genes located on BraMF2 regions in *B. rapa* genome. Bra022802 were highly expressed in the stem, but Bra022803 were highly expressed in the root. For the five members of the CYP73 gene family in *B. rapa*, the WGD- and TD-type P450s displayed different expression patterns across different tissues, indicating functional divergence among five P450s of the CYP73 gene family in *B. rapa*. In *B. oleracea*, the WGD-type P450s (Bol033349, Bol006704, and Bol004610) displayed high expression in the root and stem but reduced expression in the leaf and flower. The TD-type P450s (Bol033347 and Bol004608) showed completely different expression levels with WGD-type P450s in *B. oleracea*. For example, Bol033347 was highly expressed in the root and lowly expressed in the stem, but Bol004608 was highly expressed in the leaf and lowly expressed in the stem, which were different with expression patterns of their other member of two-copy tandem array in the WGD-type P450s from CYP73 gene family (Fig. 5b)*.* From comparison, *A. thaliana* P450 gene (AT2G30490.1) indicated different expression patterns with its corresponding homologous P450 genes in *Brassica* species. The expression patterns of the members of the CYP73 gene family in *B. rapa* were the same as those in *B. oleracea*. The WGD-type P450s displayed the same expression trends and showed completely different expression with the TD-type P450s in *Brassica* species. Among the TD-type P450s, anyone P450 gene showed a different expression with the other P450 genes in the four tissues in *Brassica* species. These results suggest that the TD-type P450s show more functional divergence compared with the WGD-type P450s in *Brassica*.

### Selection forces on P450 orthologs in *A. thaliana* and *Brassica* species

The Ka/Ks ratio was measured to investigate the strength of selection pressure for each branch of the phylogenetic tree using PAML [38]. According to the syntenic relationships between the *A. thaliana* and *Brassica* genomes, 124 and 118 *A. thaliana* P450s have 187 and 177 orthologous genes in *B. rapa* and *B. oleracea*, respectively. In total, 187 and 177 orthologous gene pairs in *A. thaliana*–*B. rapa* and *A. thaliana*–*B. oleracea* lineages were used to investigate the differential selection patterns in *A. thaliana* compared with the *B. rapa* and *B. oleracea* genomes. The mean Ka/Ks ratio of all orthologous gene pairs was 0.194 in *B. rapa* compared with the *A. thaliana* genome, which is greater than that of *B. oleracea* compared with the *A. thaliana* genome (0.191), although the difference was not significant (Mann–Whitney U-test, *P* = 0.7988 > 0.05). These results suggest that the P450 orthologous gene pairs in *A. thaliana*–*B. rapa* and *A. thaliana*–*B. oleracea* lineages underwent negative selection, but the two displayed no significant difference after *Brassica* species diverged from a common ancestor with *A. thaliana*.

To investigate the selection pressure of P450 orthologous gene pairs for intragenome in *Brassica*, the P450 gene pairs located on triplicated genomic regions in *Brassica* genomes compared with *A. thaliana* were employed to detect the difference in selection pressure between different subgenomes compared with the *A. thaliana* genome. A total of 83, 56, and 48 gene pairs in *A. thaliana* compared with the BraLF, BraMF1, and BraMF2 subgenomes in *B. rapa* were used to calculate the Ka/Ks values and evaluate the difference in P450 gene pairs among the *A. thaliana*–BraLF, *A. thaliana*–BraMF1, and *A. thaliana*–BraMF2 subgroups in the *A. thaliana*–*B. rapa* lineage, respectively. The mean Ka/Ks ratio of the P450 gene pairs was 0.189 in the *A. thaliana*–LF subgroup, which was larger than that of the *A. thaliana*–MF1 subgroup (0.188) and smaller than that of the *A. thaliana*–MF2 subgroup (0.21). These results indicate that the P450 gene pairs in the different subgenomes in *B. rapa* underwent negative selection compared with those in *A. thaliana*, but the difference was not significant (Mann–Whitney U test, P_BraLF:BraMF1_ = 0.6322 > 0.05; P_BraLF:BraMF2_ = 0.355 > 0.05; P_BraMF1:BraMF2_ = 0.2724 > 0.05). For the P450 orthologous gene pairs in *A. thaliana* compared with the different subgenomes in *B. oleracea*, 79, 56, and 43 orthologous gene pairs were detected in the *A. thaliana*–BolLF, *A. thaliana*–BolMF1, and *A. thaliana*–BolMF2 subgroups in the *A. thaliana*–*B. oleracea* lineage. Slight differences were found in their mean Ka/Ks ratios (BolLF: 0.1899, BolMF1: 0.1908, BolMF2: 0.1920), which also showed no significant differences between them (Mann–Whitney U test, P_BolLF:BolMF1_ = 0.6843 > 0.05; P_BolLF:BolMF2_ = 0.7372 > 0.05; P_BolMF1:BolMF2_ = 0.913 > 0.05). A similar inference of negative selection with the consensus evolutionary pattern for P450 gene pairs was detected in *A. thaliana* compared with the different subgenomes in *B. rapa* and *B. oleracea*, although the P450 gene pairs were distributed in different *Brassica* genomes compared with the *A. thaliana* genome.

## Discussion

### Comparison of P450 gene families in *A. thaliana* and *Brassica* lineage

On the basis of the syntenic relationships between subgenomes of *A. thaliana*, duplicated gene pairs from the α, β, and γ WGD events were identified in the *A. thaliana* genome. Retained ancient genes in different evolutionary periods can be identified by using the ancient relationship evidence from the three rounds of WGD events in the evolutionary history of angiosperms. On the basis of the syntenic relationships in *A. thaliana*, 1, 3, and 11 duplicated gene pairs were identified from the γ, β, and α WGT or WGD events to trace the ancient cytochrome P450 gene families. As mentioned above, one duplicated gene pair that was retained from the γ WGD event was the more ancient P450 duplicated gene pair in *A. thaliana*. These two ancient P450 genes were members of the CYP94 gene family, which meant that the CYP94 gene family was relatively more ancient than the P450 gene family. CYP51 is an ancient gene family in lower monocellular eukaryotes [9]. According to the classification criteria of cytochrome P450 nomenclature by Dr. Nelson [45], the CYP51 gene family was distributed in Part C (covers CYP10–CYP69 and 501–699 and 5001 and higher), which included the P450 gene families identified in animals, fungi, and other lower eukaryotes [4]. Except for CYP51, all P450 gene families in the present study were distributed in Part D (covers CYP71–CYP99 and 701–772), which only contained cytochrome P450 gene families in plants. After detecting duplicated gene pairs from the three rounds of WGD in *A. thaliana*, members of the CYP51 gene family were not detected, indicating that the duplicated genes of the CYP51 gene family were quickly lost after each WGD event in angiosperms. This phenomenon may be the reason why only one P450 gene was detected in the CYP51 gene family on the basis of the TAIR10 version, although this P450 gene family was older than other gene families. A total of 3 and 11 duplicated gene pairs were detected from the β and α WGD events in *A. thaliana*, which covered 3 and 11 P450 gene families for the two WGD events, respectively. However, members of the CYP94 gene family in the 14 gene families were not found, suggesting the loss of the gene pairs of CYP94 in the latter two WGD events. For the latter two WGD events, only two (CYP81 and CYP86) of the four P450 gene families retained in the β WGD event were examined in the α WGD event. Thus, the rest of the gene family was lost after the α WGD event. Finally, 13 P450 gene families were influenced by the three rounds of WGD events in angiosperms. The gene pairs of the remaining P450 gene families possibly experienced quick loss or the members of P450 gene families later emerged in *A. thaliana* after three rounds of WGD in angiosperms.

Taking the P450 gene families in *A. thaliana* as reference, 47 P450 gene families in *A. thaliana* retrieved 46 corresponding P450 gene families in each *B. rapa* and *B. oleracea*, and the CYP716 gene family was lost in two *Brassica* species. Members of the CYP716 gene family are involved in brassinosteroid biosynthesis, brassinosteroid homeostasis, multicellular organismal development, and sterol metabolism [46]. We speculated that the loss of the CYP716 gene family *B. rapa* and *B. oleracea* might indicate the incomplete genome sequencing and assembling for the two *Brassica* species. Moreover, there were four specific subgroups compared to *A. thaliana* detecting through phylogenetic analysis, which referred to one *B. rapa* and five *B. oleracea* P450s. These six cytochrome P450s were grouped into five different clans of cytochrome P450 gene families. Analysis of protein sequences of these six P450s revealed that these P450s contained partial sequences of p450 conserved domain of standard P450 protein. This result might indicate the incomplete gene prediction or gene function loss of P450s due to selection pressure.

### Family-specific evolution of P450 gene families in *Brassica*

The *Brassica* lineage underwent WGT and generated triplicated genomic regions in *B. rapa* and *B. oleracea* after the *Brassica* lineage split from a common ancestor with *A. thaliana*. According to the syntenic relationships between the *A. thaliana* and *B. rapa* genomes, the orthologous P450 genes of 124 and 118 *A. thaliana* P450s were detected 187 *B. rapa* P450s and 177 *B. oleracea* P450s, which were distributed in 41 and 42 P450 gene families in *B. rapa* and *B. oleracea*, respectively. Compared with the 46 P450 gene families in *Brassica* species, five and four P450 gene families in *B. rapa* and *B. oleracea* have not detected syntenic orthologous P450s in *A. thaliana*, indicating the loss of duplicated genes of the remaining P450 gene families after the WGT in the *Brassica* lineage. Through the analysis of TD for the P450 supergene family, tandem duplicated genes for cytochrome P450s were identified (132/251, 52.59% in *A. thaliana*; 137/354, 38.7% in *B. rapa*; and 112/343, 32.65% in *B. oleracea*) in *A. thaliana* and *Brassica* species. Comparison of influence of the WGD and TD events for the P450 supergene family between *A. thaliana* and *Brassica* species indicated that TD exerted more effect on the evolution of the P450 supergene family in *A. thaliana* than in *Brassica* species, and WGD showed more influence than TD on the evolution of the P450 supergene family in *Brassica* lineage. Although previous study revealed that genes with essential functions are less duplicated across evolution [47], these two evolution mechanisms still caused the increase of the members of P450 gene families in *A. thaliana* and *Brassica* lineage, leading to the 1.4 times members of P450 gene families in *Brassica* species.

According to the expansion or reduction rates of P450 gene families, 43 P450 gene families were influenced by WGD or TD in *A. thaliana*, *B. rapa*, and *B. oleracea*. The remaining P450 gene families were not detected in any of the WGD-type and TD-type P450s in *A. thaliana* and *Brassica* species. These results suggested that not all P450 gene families were influenced by WGD and TD, although the two events played an important role in the evolution of the P450 supergene family in *A. thaliana* and *Brassica* lineage. After comparing the different evolution mechanisms of P450 gene families between *A. thaliana* and *Brassica* species, we can conclude that the evolution of each P450 gene family in *Brassica* species has its own family-specific mechanisms.

### Dynamics of TD in the evolutionary history of the P450 gene family

According to syntenic relationships between *A. thaliana* and *Brassica* species, tandem duplicated genes were classified to confirm ancient tandem duplicated genes through analysis of location on syntenic genomic regions in *A. thaliana* compared with *B. rapa* and *B. oleracea* genomes. This result indicates that these ancient tandem duplicated genes were generated before the split of *A. thaliana* and *Brassica* ancestor from a common ancestor. Except for the ancient tandem duplicated genes, the remaining tandem duplicated genes in *A. thaliana* and *Brassica* lineage have not showed ancient syntenic relationships. This result indicates that these genes were generated after divergence of the *Brassica* ancestor. The cytochrome P450 genes that formed after the divergence of *Brassica* ancestor were species-specific tandem duplicated genes in *B. rapa* and *B. oleracea*. These results suggest that the tandem duplicated P450 genes formed in a continuous historical process, ending with the evolutionary history of cytochrome P450s. This phenomenon is consistent with a previous report that tandem duplication occurred continuously throughout the evolutionary history of corresponding species [42].

## Conclusions

The cytochrome P450 gene superfamily is involved in the biosynthesis of various primary and secondary metabolites. This study identified 251, 354, and 343 P450 genes in *A. thaliana*, *B. rapa*, and *B. oleracea*, respectively. These P450 genes were classified into 47 P450 gene families. Evolutionary analysis of P450s in different organisms revealed that 948 P450s of P450 gene families were grouped into non-A-type and A-type classes among the three species. Chromosomal organization and composite phylogenetic analysis provided a clear distribution on the chromosomes and evolutionary relationship of cytochrome P450 genes among *A. thaliana*, *B. rapa*, and *B. oleracea*. The inferred phylogeny and syntenic relationships of the P450 genes between *A. thaliana* and *Brassica* species indicated that the family-specific evolution in the *Brassica* lineage can be attributed to both WGD and TD, whereas WGD was recognized as the major mechanism for the recent evolution of the P450 supergene family in the *Brassica* lineage. Expression analysis of the members of P450 gene family in *A. thaliana* and *Brassica* species revealed that the WGD-type P450s showed the same expression pattern, but the TD-type P450s showed completely different expression across different tissues in *A. thaliana* and *Brassica* species. The WGD- and TD-type P450s showed different expression patterns in the four tissues in *A. thaliana* and *Brassica* species. Selection force analysis suggested that the P450 orthologous gene pairs in *A. thaliana* compared with *Brassica* species underwent negative selection, but no significant differences were found between orthologous P450 gene pairs in the *A. thaliana*–*B. rapa* and *A. thaliana*–*B. oleracea* lineages. The P450 orthologous gene pairs in the different subgenomes in *B. rapa* or *B. oleracea* compared with *A. thaliana* also underwent negative selection, but Mann-Whitney U test results indicated no significant differences among them. Through comparative analysis of P450 genes among *A. thaliana*, *B. rapa*, and *B. oleracea*, we investigated the influence of WGD and TD on the evolutionary history and functional divergence of P450 gene families in the genus *Brassica*. This study provides a biology model to study the mechanism of gene family formation, particularly in the context of the evolutionary history of angiosperms, and offers novel insights for the study of angiosperm genomes.

## Additional files

Additional file 1: Table S1. Members of cytochrome P450 gene families in *A. thaliana*, *B. rapa*, and *B. oleracea*. The basic information of cytochrome P450s in *A. thaliana*, *B. rapa*, and *B. oleracea* contained the name of gene family, the location of conserved domains and protein sequences. (XLSX 298 kb)

Additional file 2: Figure S1. Distribution of cytochrome P450 genes in *A. thaliana*, *B. rapa* and *B. oleracea*. A. Chr1-Chr5 represent pseudochromosomes in *A. thaliana*. B. A01–A10 represent pseudochromosomes in *B. rapa*. C. C01–C09 represent pseudochromosomes in *B. oleracea*. Green bars represent pseudochromosomes of three species. Balck lines on pseudochromosomes represent the location of P450s on pseudochromosomes in *A. thaliana*, *B. rapa* and *B. oleracea*. (JPEG 6260 kb)

Additional file 3: Figure S2. Phylogenetic analysis of cytochrome P450 gene families in *A. thaliana* and *Brassica* species. I and II represent different groups among three species. Red solid circles represent cytochrome P450 genes in *A. thaliana*, pink solid circles represent cytochrome P450 genes in *B. rapa*, and blue solid circles represent cytochrome P450 genes in *B. oleracea*. CYP represents cytochrome P450 gene family. *B. rapa*-specific represents specific cytochrome P450 genes in *B. rapa*. *B. oleracea*-specific represents specific cytochrome P450 genes in *B. oleracea*. *Brassica*-specific represents specific cytochrome P450 genes in *Brassica* species. (JPEG 886 kb)

Additional file 4: Table S2. Orthologous genes pairs from different whole genome duplication events in *A. thaliana* compared with different subgenomes in *B. rapa* and *B. oleracea*. This file contained the orthologous genes pairs of cytochrome P450s in *A. thaliana* compared to BraLF, BraMF1, and BraMF2 subgenomes in *B. rapa*, and BolLF, BolMF1, and BolMF2 subgenomes in *B. oleracea*, respectively. (XLSX 24 kb)

Additional file 5: Table S3. Tandem arrays of cytochrome P450 genes among *A. thaliana*, *B. rapa*, and *B. oleracea*. The basic information of tandem arrays of cytochrome P450s among three species included the gene family, location, name, gene number, and gene list of tandem arrays. (XLSX 31 kb)

Additional file 6: Table S4. Expression values of cytochrome P450 genes across different tissues among *A. thaliana*, *B. rapa*, and *B. oleracea*. This file contained the expressed cytochrome P450s of *A. thaliana* in root, stem, leaf, and flower tissues according to the analysis of microarray data, and the expressed cytochrome P450s of *B. rapa, and B. oleracea* in root, stem, leaf, and flower tissues according to the analysis of RNA-seq data. (XLSX 72 kb)

Additional file 7: Table S5. Expression statistics of the members of cytochrome P450 gene families among *A. thaliana*, *B. rapa*, and *B. oleracea*. This file contained the proportion of expressed P450s in different cytochrome P450 gene family among three species. (XLSX 12 kb)

## Abbreviations

HMM: Hidden Markov model
ML: Maximum likelihood
Mya: Million years ago
TD: Tandem duplication
WGD: Whole genome duplication
WGT: Whole genome triplication
