# Supplementary figures and images for: Evolutionary history and functional divergence of the cytochrome P450 gene superfamily between Arabidopsis thaliana and Brassica species uncover effects of whole genome and tandem duplications
Source: BMC Genomics. 2017 Sep 18;18:733. doi: 10.1186/s12864-017-4094-7 (PMC5604286; doi:10.1186/s12864-017-4094-7)

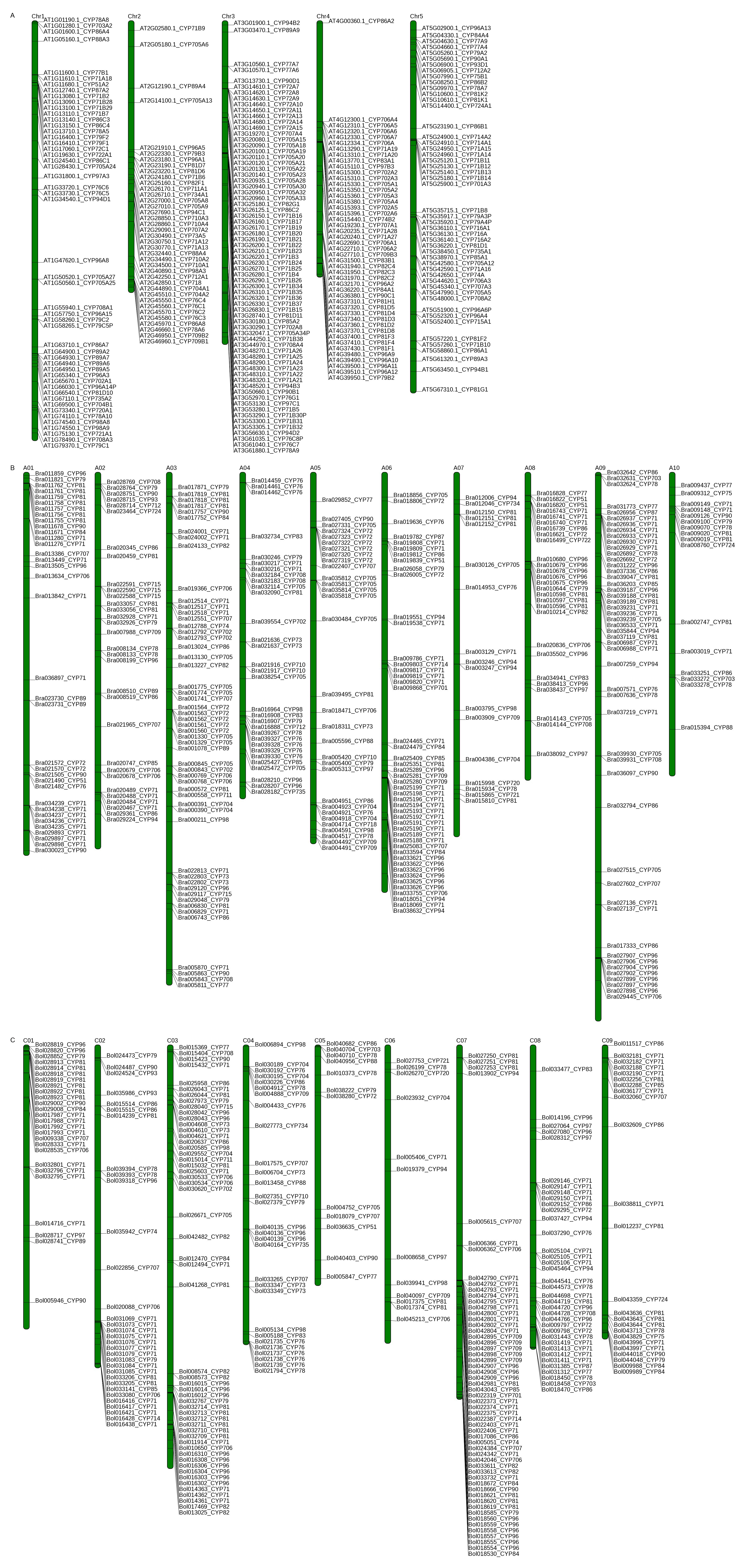

Supplement: Supplementary file 2 — Distribution of cytochrome P450 genes in A. thaliana, B. rapa and B. oleracea. A. Chr1-Chr5 represent pseudochromosomes in A. thaliana. B. A01–A10 represent pseudochromosomes in B. rapa. C. C01–C09 represent pseudochromosomes in B. oleracea. Green bars represent pseudochromosomes of three species. Balck lines on pseudochromosomes represent the location of P450s on pseudochromosomes in A. thaliana, B. rapa and B. oleracea. (JPEG 6260 kb) [file 12864_2017_4094_MOESM2_ESM.jpg]

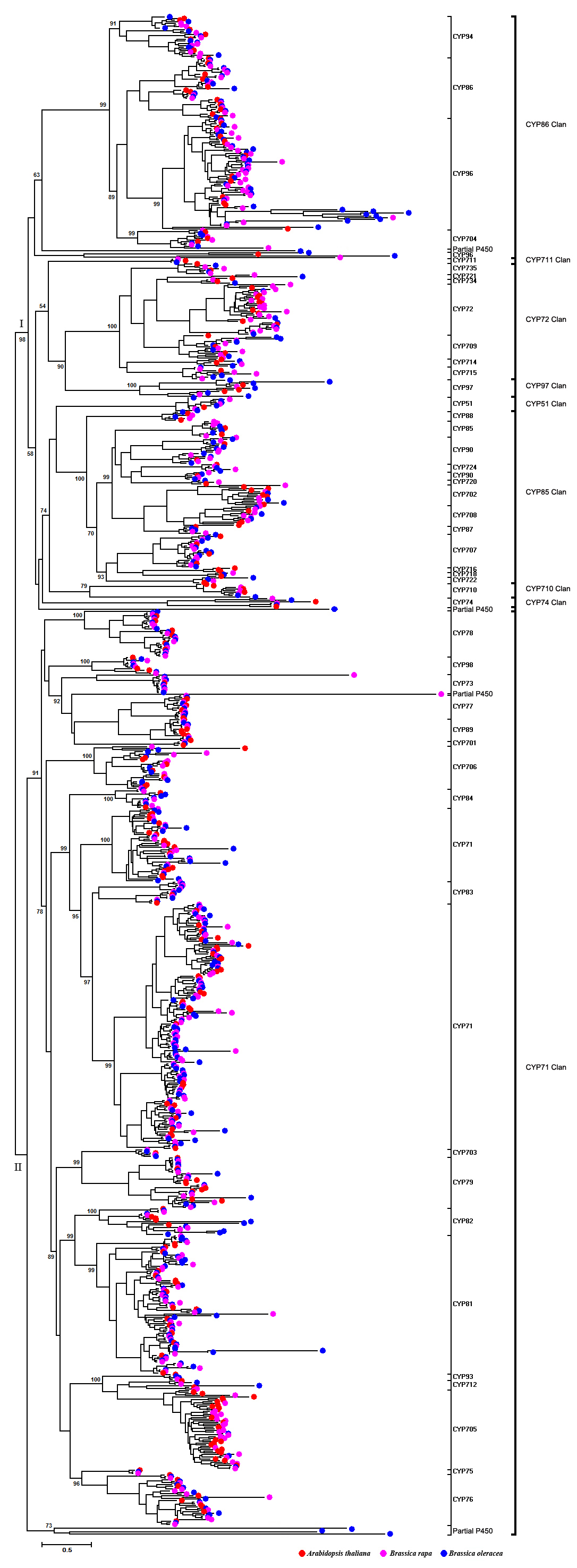

Supplement: Supplementary file 3 — Phylogenetic analysis of cytochrome P450 gene families in A. thaliana and Brassica species. I and II represent different groups among three species. Red solid circles represent cytochrome P450 genes in A. thaliana, pink solid circles represent cytochrome P450 genes in B. rapa, and blue solid circles represent cytochrome P450 genes in B. oleracea. CYP represents cytochrome P450 gene family. B. rapa-specific represents specific cytochrome P450 genes in B. rapa. B. oleracea-specific represents specific cytochrome P450 genes in B. oleracea. Brassica-specific represents specific cytochrome P450 genes in Brassica species. (JPEG 886 kb) [file 12864_2017_4094_MOESM3_ESM.jpg]
